# Evidence for the Encoding of a Motion's Goal in the Monkey Brain

**DOI:** 10.1371/journal.pbio.0040169

**Published:** 2006-04-25

**Authors:** Françoise Chanut

From cooking to playing the piano, our activities consist of simple motions that we string together for specific purposes. When we learn a new recipe or piano sonata, we memorize the elemental gestures of the trade as well as the precise order in which they must occur to produce a coq au vin or “Moonlight Sonata.” And when we start cooking or playing, we somehow summon the sequence of movements that will lead to our goal.

Scientists have long wondered how the brain stores in memory a sequence of movements. Ultimately, such memories result from connections that brain cells (neurons) establish among themselves and with the relevant muscles during the learning period. But precisely how the information is organized in the brain remains largely mysterious. A model called “associative chaining” proposes that a memory cell representing an early motion activates memory cells representing later motions in the sequence. In this model, a memory cell recalls at once the type of motion and its order of appearance in the sequence. But in a new study, Mark H. Histed and Earl K. Miller show that they can uncouple the memories for a sequence's components and for their serial order by manipulating a subset of cells in the brain of monkeys. Their observations suggest the existence of abstract forms of memory that store the goal of a sequence of movements.

The researchers focused on a small area near the surface of the brain's frontal lobe called the supplementary eye field (SEF). Within the SEF are cells that control small voluntary eye movements called saccades that animals use to track bright objects near the periphery of their field of vision. Stimulation of individual SEF neurons triggers saccades to separate locations, and SEF neurons have been found to fire sequentially while monkeys made serial saccades to visual targets, as if the cells controlled the saccade succession. The SEF therefore appeared like a logical place to look for neurons that harbor memories of learned saccade sequences.

Histed and Miller first trained two monkeys to saccade to successive locations on a screen. While the monkeys focused on a bright central spot, two additional spots (cues) would appear at random one after the other and disappear after half a second. The monkeys had to wait one more second before saccading to the two cued locations in the order of the cues' appearance. The task therefore required memorizing both the cues' location and order. The monkeys were rewarded each time they accomplished a saccade sequence correctly, and they eventually performed with a near-perfect score. In an experimental session, the researchers would stimulate various sites in the SEF with microelectrodes during the one-second delay period, and record the effect of these stimulations on the monkeys' performance.

Out of 55 SEF locations tested, the researchers found 25 that disrupted the monkeys' performance. The pattern of errors was always the same: the monkeys saccaded to the correct locations, but in incorrect order. For instance, if the sequence consisted of a cue at a 1 o'clock location followed by a cue at 3 o'clock, the monkeys would saccade to 3 o'clock first and then 1 o'clock. However, they never saccaded to 5 o'clock or 11 o'clock, or to any other wrong location. Therefore, the monkeys seem able to keep the memory of visual cues intact even when they forget the order of the cues' appearance, which shows that memories for the order versus the components of a sequence are held separately in the monkey's brain. More specifically, these results indicate that the SEF can reorganize a series of saccades.

Strong stimulation of the SEF can elicit eye movements. If you vertically divide the visual world in half, stimulating the right SEF causes saccades to the left field, and vice versa. But the stimuli Histed and Miller applied to the SEF were too weak to produce saccades. Nevertheless, their effects were similarly biased: they reordered saccade sequences such that the monkeys always seemed to aim for left field when receiving a stimulus in the right SEF, and to right field when receiving a left stimulus. The researchers conclude that rather than precisely ordering a sequence of saccades, the SEF codes for a spatial goal (left field or right field), regardless of whether it takes one or more saccades to reach that goal.

The implication of these results is that the brain might store complex sequences of movements in two forms: one for the sequence's goal and one for the details of its execution.

## 

**Figure:**
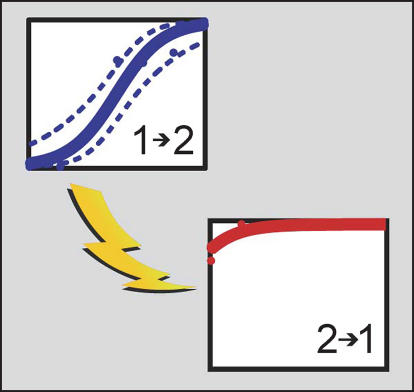
The sequential order of short-term memory can be reversed by using tiny amounts of electrical current to change neural activity in the frontal lobe of the brain.

